# A systematic review of the indirect costs of schizophrenia in Europe

**DOI:** 10.1093/eurpub/cky231

**Published:** 2018-11-05

**Authors:** A Fasseeh, B Németh, A Molnár, F-U Fricke, M Horváth, K Kóczián, Á Götze, Z Kaló

**Affiliations:** 1 Syreon Research Institute, Budapest, Hungary; 2 Eötvös Loránd University (ELTE), Budapest, Hungary; 3 Technische Hochschule Nürnberg, Nürnberg, Germany; 4 Gedeon Richter Plc., Budapest, Hungary

## Abstract

**Background:**

Schizophrenia is a chronic disease associated with significant and long-lasting effects on health, and it is also a social and financial burden, not only for patients but also for families, other caregivers, and the wider society. It is essential to conduct the assessment of indirect costs, to understand all the effects of the disease on society. Our aim is to gain a better understanding of the indirect costs of schizophrenia in Europe.

**Methods:**

We conducted a comprehensive systematic literature review covering EMBASE, Medline, and PsycINFO as well as reviewing Health Technology Assessment databases from different countries. We used a qualitative research synthesis for presenting information, as most of the studies were methodologically diverse, a quantitative analysis would have been impractical.

**Results:**

Indirect cost adjusted to inflation ranged vastly between studies included in the review from 119 Euros to 62, 034 Euros annually. The average proportion of indirect costs of total costs was 44%. Studies highlighted important cost drivers as age, gender, and disease severity, explaining the variation in costs between treatment and patient groups.

**Conclusions:**

Regardless of the methodological heterogeneity of the reviewed studies, there was an agreement about the significance of indirect costs of schizophrenia on the society. Considering the relatively high prevalence of schizophrenia in Europe, a need for more cost of illness studies especially from Central Eastern and Southern Europe is suggested.

## Introduction

Schizophrenia is a disorder of the brain which affects how a person thinks, feels, and perceives reality^1^ and significantly decreases the quality of life of patients.^2^ In the European Union population, the estimated prevalence of all psychotic disorders is around 1.2%, and the incidence of schizophrenia is 15.2 per 100 000 persons. People with schizophrenia have 2 to 3 times higher risk of death compared to the general population,^3^ and the lifetime risk of schizophrenia morbidity is 7.2 per 1000 persons.^4^ This disease is also associated with the social stigmatization of patients.^5^

Individuals with schizophrenia use a substantial amount of healthcare services. This condition imposes a significant economic burden on both the patients and their families, and on the society as a whole.^6^ The quantifiable costs associated with human diseases and illness are typically categorized into two unique components, direct and indirect cost components, the general focus in the scientific literature is on direct cost component. However, to conduct thorough analyses on the effects of schizophrenia on society, the assessment of indirect costs is equally important.^7^ In published cost-effectiveness analyses, many analysts continue to claim a societal perspective, while they collect and analyse data only from a payer perspective.^8^

Aside from the stress and decline in the quality of life, caregiving acts as an independent risk factor for mortality of schizophrenia.^9^ Moreover, caregiver’s productivity is affected as they often have to cut back on their working hours, take a leave of absence and may receive a warning about performance or attendance.^10^ At the same time, informal care is increasingly being considered as a valuable substitute and complement of expensive formal care.^11^

The objective of this study was to identify and review the most recent evidence on the extent of the indirect cost of schizophrenia in Europe, and the factors that influence these costs taking into consideration both the requirements of HTA agencies preferring up-to-date data and providing sufficient information to draw a general conclusion. This research particularly focused on; (i) the published evidence on indirect costs and caregiver burden associated with schizophrenia; (ii) extent of the total indirect cost related to schizophrenia; (iii) the proportion of indirect costs compared to total costs of schizophrenia; (iv) the most important factors associated with the variations in indirect costs and caregiver burden of schizophrenia.

## Methods

A systematic literature review was conducted, and the results were presented using qualitative evidence synthesis. Indirect cost as a percentage of total cost of schizophrenia was calculated to give an idea on how the indirect cost relates to the total cost of disease.

### Databases and literature search strategy

The literature search was performed on 30 March 2017, on the following databases: MEDLINE (Medical Literature Analysis and Retrieval System Online) (via Scopus), EMBASE (Excerpta Medica dataBASE) (via Scopus) and PsycINFO (via Ovid). The WHO HEN (World Health Organization Health Evidence Network), NHS (National Health Service) (United Kingdom), IQWiG (Institut für Qualität und Wirtschaftlichkeit im Gesundheitswesen) (Germany), AQUAS (Agència de Qualitat i Avaluació Sanitàries de Catalunya) (Spain), SBU (Statens Beredning för Medicinsk Utvärdering), and Sahlgrenska Universitetssjukhuset (Sweden), and the NHS CRD (National Health Service Centre for Reviews and Dissemination) (United Kingdom) Health Technology Assessment databases were also searched for relevant studies.

The search term was constructed as a combination of domains related to ‘indirect cost’ and ‘schizophrenia’ (see Supplementary Appendix). The literature search was limited to English language papers published since 2011 till 30 March 2017, searching in the title, abstract, and keywords of the articles in Scopus, and the title, abstract, heading words, table of contents, key concepts, original title, and the tests and measures in Ovid.

We only considered relatively recent papers published after the year 2011. Our aim was to balance between the requirements of HTA agencies preferring up-to-date data and to have sufficient information to draw a conclusion. When the initial date for inclusion was selected, we considered that major policy and treatment changes with potentially significant impact on indirect health care cost, including deinstitutionalization of patients, shift toward generic and/or long-acting injectable drugs were implemented earlier than 2011. Hence the period since 2011 could reasonably be considered a fairly homogenous period in the management of schizophrenia.

Due to the overlap between the databases, search results were first de-duplicated using the embedded feature of EndNote software version X7.5, and any other duplication was checked during the title and abstract screening as well as during the full-text review. The title- and abstract-based screening were conducted by two independent reviewers; any disagreements were resolved by a third, principal researcher. Although our review focused on Europe, no country restriction was applied during the literature search phase, as we were concerned about losing potentially relevant papers not labeled to contain data from a European country, instead papers not reporting any data on European countries were excluded during the title-abstract screening phase and full-text review. Furthermore, papers cited in systematic literature reviews (SLRs) were identified, and in case of eligibility, the pool of included papers was extended.

### Title and abstract screening

As a first step, titles and abstracts of all articles were screened using pre-defined exclusion criteria to exclude:

1. Papers without an English abstract.
2. Book sections.
3. Papers clearly stating that study is concerned only with a non-European geographical region.
4. Papers not focusing on schizophrenia or studies considering not only schizophrenic patients (e.g. the dealing with the indirect costs of mental illnesses in general).
5. Papers not describing a systematic or targeted literature review, meta-analysis, or a human observational or experimental study.
6. Studies with sample size lower than 50 patients and in which results are based on primary data collection from the sample.
7. Papers not reporting data relevant to the research topic (i.e. the paper does not provide data on indirect costs).

### Data extraction

As a second step, papers which met the above-mentioned screening criteria were reviewed in full text to check eligibility for data extraction. Standardized data extraction form was developed and assessed for suitability. A pilot data extraction form was circulated among all reviewers, and the extraction grid was finalized according to the comments of the reviewers. Screened papers were excluded if (1) there was no English full-text version available; (2) the study was completed before 2006.

All extracted data were double checked by another researcher. The result of this process formed the basis of the qualitative evidence synthesis.

### Assessment of methodological quality of included studies

Quality assessment of the studies was performed using three different methods (depending on the type of study). Two cost-effective analysis studies were evaluated using the CHEERS checklist^12^ both scoring 100%. Also, two literature reviews were evaluated using the PRISMA checklist^13^ with score of 81% and 88% with the major issue in methodology. On the other hand, cost of illness (COI) studies were evaluated using the checklist developed by Larg et al.^14^ and the average score was 73%, while individual papers scores ranged from 61–86%.

### Cost adjustment

To allow comparability of the results, costs were adjusted to the pricing year 2016. For inflation, consumer price index (CPI) data provided by the World Bank^15^ were used. In addition to the local currency, values were expressed in Euros using the yearly currency exchange rate for the year 2016.^16^

Following the definition of the German Institut für Qualität und Wirtschaftlichkeit im Gesundheitswesen,^17^ in this paper only the loss of leisure time was considered in the direct non-medical cost domain, while productivity loss by patients or caregivers was considered as an indirect cost.

### Productivity and caregivers

Productivity loss of patients due to morbidity or mortality was defined using two primary approaches. The human capital approach (HCA) aims to reflect productive loss potential by multiplying the loss earnings for different age and sex groups by the corresponding number of patients in that group. The friction cost approach (FCA) assumes that patients who stop working because of illness will be replaced by someone who was previously unemployed and therefore, measures only the productivity loss during the time required to replace a worker.^18^ FCA is relatively difficult to implement as it would require detailed information on the labor market conditions and behaviors.

The indirect cost of schizophrenia can be broken down to productivity loss by patients and productivity loss by caregivers (informal care).^19^ Formal caregivers are paid to provide care in one’s home or in a care setting (daycare, residential facility, and long-term care facility). Informal caregivers are unpaid individuals (e.g. a spouse, partner, family member, friend, or neighbor) involved in assisting others with activities of daily living and/or medical tasks.^10^

## Results

**Figure 1:**
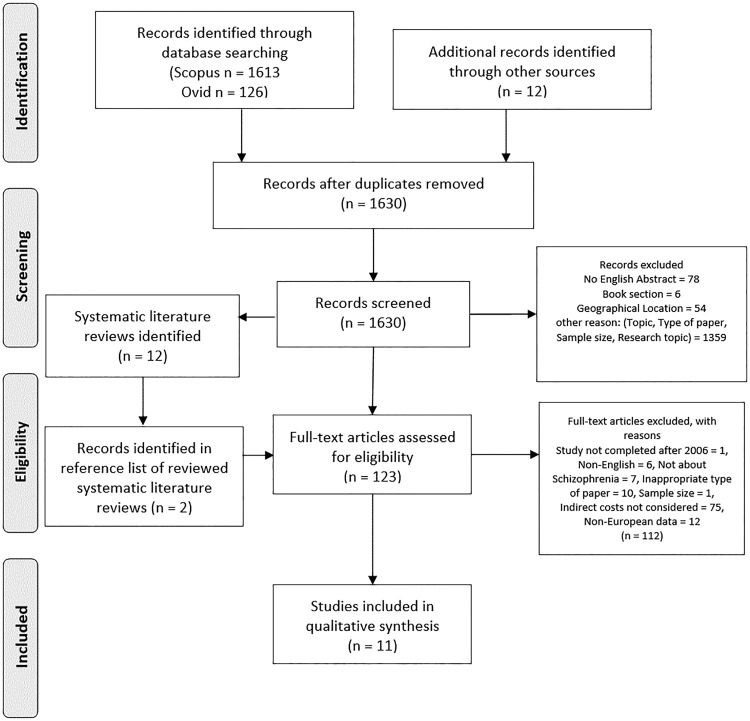
Flow diagram of the systematic literature search

One-hundred and twenty-three studies were found to be eligible for full-text review: 121 out of the 1630 screened abstracts were included, and other 2 papers were identified in the reference lists of the reviewed SLRs. Figure 1 illustrates the literature selection process.

As a result of the full-text review, 11 studies were included in the qualitative synthesis, from which 7 studies had a comparative design and 4 had non-comparative design. Two articles presented results for the subgroup of patients with predominantly negative symptoms (PNS).

Eight studies adopted the societal perspective only, while three papers considered more than one perspective. From the 11 articles, three did not specify the disease diagnostic criteria for schizophrenia. In four studies, the diagnostic criteria of International Classification of Diseases (ICDs) were applied, two papers used the Operational Criteria Checklist for Psychotic Illness and Affective Illness (OPCRIT)^20^ classification, while two studies performed patient inclusion based on the Diagnostic and Statistical Manual of Mental Disorders-IV (DSM-IV). Differences were found considering the included ICD-10 codes. Two articles used the German modification of ICD-10. A summary of the characteristics of included studies is reported in table 1.

**Table 1:** Overview of objective, study design, location, and analysis year

First author (year)	Study design	Country^a^	Analysis year (follow-up period)	Study objective
**ARANDA-RENEO (2013)^21^**	Cross-sectional	ESP	2007 and 2008	Analyze the value of informal care associated with the loss of personal autonomy (dependency) caused by schizophrenia in Spain.
**BARNES (2016)^22^**	Prospective	UK	2011–2014	Test the benefits of citalopram (a SSRI antidepressant) for people with schizophrenia and negative symptoms in terms of improved quality of life and reduction of negative symptoms, as well as recording the relative risks and costs of this augmentation of antipsychotic medication.
**CORTESI (2013)^23^**	Retrospective & prospective	ITA	2006 and 2007	Assess persistence, compliance, costs, and Health-Related Quality-of-Life (HRQoL) in young patients undergoing antipsychotic treatment according to clinical practice.
**CRAWFORD (2012)^24^**	Prospective	UK	2007 and 2008	Examine the impact of referral to community-based group art therapy for people with schizophrenia compared with referral to an active control treatment or to standard care alone.
**EINARSON (2014)^25^**	Retrospective	SWE	2007–2012	Determine the cost-effectiveness of long-acting injectable (LAI) antipsychotics for chronic schizophrenia in Sweden.
**EKMAN (2013)^26^**	Retrospective	SWE	2006–2008	Investigate the healthcare resource utilization and cost-of-illness in patients with schizophrenia in Sweden and to relate the cost to hospitalizations and global assessment of functioning (GAF).
**EVENSEN (2016)^27^**	Retrospective	NOR	2012	Access national health and welfare registers covering the entire Norwegian population to establish 12-month prevalence of schizophrenic patients, 12-month employment rate, and 12-month cost of schizophrenia.
**FREY (2014)^28^**	Retrospective	GER	2005–2008	Investigate the burden of schizophrenia in Germany.
**GUPTA (2015)^29^**	Retrospective	FRA; GER; ITA; ESP; UK	2010–2013	Understand the impact of providing care for adults with schizophrenia on productivity, resource utilization, and costs in the EU5 (France, Germany, Italy, Spain, and UK).
**PLETSCHER (2015)^30^**	Retrospective	CHE	2001–2012	Estimate the prevalence of schizophrenia in Switzerland and to assess its burden on patients, caregivers, and society as a whole.
**SICRAS-MAINAR (2014)^31^**	Retrospective	ESP	2011 and 2012	Evaluate the prevalence and impact of negative symptoms on healthcare resources utilization and costs in patients with schizophrenia.

a CHE: Switzerland, GER: Germany, ESP: Spain, FRA: France, ITA: Italy, NOR: Norway, SWE: Sweden, UK: United Kingdom.

The study of data collection period ranged from 2001 to 2014. All studies presented data from Western-European countries. Seven studies had a retrospective design, two had a prospective design, while one used both, and had a cross-sectional study design. Bottom-up cost calculation approach was used by eight studies, while only one paper applied for top-down approach. The sample size of included studies ranged from 62 to 16 448 patients. Regarding age range of subjects in the included papers, Aranda -Reneo (2013) used a representative sample on the national and the regional level in Spain, Evensen (2016) included subjects of ages ranging from 15 to 67 years old, while Pletscher (2015) and Einarson (2014) did not explicitly state the age range or age criteria for inclusion in the study. Frey (2014) used the sickness fund claims database in Germany and was the only paper to evaluate the differences in cost between different age groups. Other papers included in the review explicitly mentioned inclusion of either adults, or 18+ patients.

The average reported indirect cost of schizophrenia from nine studies was 20 664 Euros (2016). The annual indirect cost of schizophrenia is presented in table 2.

**Table 2:** Annual indirect cost of schizophrenia per patient

Article	Countries	Pricing year	Number of patients	Indirect cost value	Currency	Annual indirect cost per patient (Euros 2016)	Indirect cost as a percentage of total cost	Patient/ caregiver	Detailed method
**ARANDA-RENEO 2013**	ESP	2008	234	27 199–57 494	EUR	29 347–62 034	N.A.	Caregiver	Proxy good method (how much it would cost to substitute or replace the informal caregiver by hiring a professional caregiver)
**BARNES 2016**	GBR	2013 and 2014	62	8585	GBP	10 746	62%	Patient, caregiver	1-informal care utilization 2-absenteeism for those who were employed
**EINARSON 2014**^d^	SWE	2011	N.A.	7117	SEK	764	3%	Patient	Human capital method
**EKMAN 2013**^e^	SWE	2008	2085	33 020	EUR	34 770	77%	Patient	Human capital method
**EVENSEN 2016**^c^	NOR	2012	8399	421 359	NOK	50 010	45%	Patient	Human capital method
**FREY 2014**	GER	2008	16 448	10 277	EUR	11 192	45%	Patient, caregiver	Friction cost method
**GUPTA 2015**^f^	FRA; GER; ITA; ESP; GBR	2013	398	6667	EUR	6720	N.A.	Caregiver	Human capital method
**PLETSCHER 2015**	CHE	2012	1666^b^	26 437	EUR	25 961	67%	Patient, caregiver	Friction cost method (absenteeism & presenteeism)
**SICRAS-MAINAR 2014**	ESP	2012	1120	118	EUR	119	6%	Patient	Friction cost method (temporary or permanent sick leave)

a CHE: Switzerland, GER: Germany, ESP: Spain, FRA: France, ITA: Italy, NOR: Norway, SWE: Sweden, UK: United Kingdom, EUR: Euros, GBP: Pounds Sterling, SEK: Swedish Krona, NOK: Norwegian Krone, N.A.: Not applicable.

b Estimated in the sample region.

c Estimated total national costs.

d Average costs from all arms (different treatment scenarios) were used because costs for general population were not reported.

e Cost of Schizophrenia (not including community mental health care).

f In case of Gupta (2015), France has been used as the reference country for adjusting prices. In case of Barnes (2016), the year 2013 was considered as the pricing year as in the paper they reported pricing year as 2013 and 2014.

The average share of indirect cost was 44%, while papers that included both the cost of productivity loss by patients and by caregivers had a higher mean of 58% (see table 2). Indirect cost adjusted to inflation ranged vastly between studies, from 119 to 62 034 Euros.

Factors associated with the higher indirect cost of schizophrenia were identified in the reviewed studies. Papers summarized in table 3 reported difference in indirect costs based on patient characteristics, as age, sex and severity of symptoms. The strongest predictor of cost was the severity of symptoms that was reported to cause 2.5-fold increase in cost for the most severe cases.

**Table 3:** Factors associated with incremental indirect costs schizophrenia among different patient groups

Cost determinant	Investigated factor	Comparator	Difference in cost between subgroups (Euros/patient/year)	Rate	Country	Reference, first author (year)
**Age**^a^	Age <=25 indirect cost (productivity loss by patient) attributable to schizophrenia^a^	Average indirect cost (productivity loss by patient) attributable to schizophrenia in the total population	−978	−38%	Germany	Frey (2014)
	Age 26–45 indirect cost (productivity loss by patient) attributable to schizophrenia^a^		127	5%	Germany	Frey (2014)
	Age 46–65 indirect cost (productivity loss by patient) attributable to schizophrenia^a^		747	29%	Germany	Frey (2014)
	Age <=25 indirect cost (informal care) attributable to schizophrenia^a^	Average indirect cost (informal care) attributable to schizophrenia in the total population	743	11%	Germany	Frey (2014)
	Age 26–45 indirect cost (informal care) attributable to schizophrenia^a^		46	1%	Germany	Frey (2014)
	Age 46–65 indirect cost (Informal care) attributable to schizophrenia^a^		−195	−3%	Germany	Frey (2014)
	Age <=25 indirect cost (total) attributable to schizophrenia^a^	Average indirect cost (total) attributable to schizophrenia in the total population	−235	−2%	Germany	Frey (2014)
	Age 26–45 indirect cost (total) attributable to schizophrenia^a^		173	2%	Germany	Frey (2014)
	Age 46–65 indirect cost (total) attributable to schizophrenia^a^		552	6%	Germany	Frey (2014)
**Sex**	Mexen	Women	6425	22%	Sweden	Ekman (2013)
**Severity of symptoms**	GAF score 50–69 (mild to serious symptoms)	GAF score >=70 (no or slight symptoms)	15 207	103%	Sweden	Ekman (2013)
	GAF score <50 (serious symptoms to severe impairment)	GAF score >=70 (no or slight symptoms)	22 373	151%	Sweden	Ekman (2013)
	Outpatients with negative syndrome	Outpatients without negative syndrome	22.7	21%	Spain	Sicras-Mainar (2014)

a The average treatment effect for the treated (ATT) equals excess resource use attributable to schizophrenia.

b GAF: global assessment function.

## Discussion

Despite the observed methodological heterogeneity and variation in cost components, there was an agreement between study results about the significance of the indirect cost of schizophrenia on the society. There were significant differences between indirect costs of schizophrenia reported by different studies. The magnitude of variability can be explained by the finding that different indirect cost elements are evaluated, different methodologies for evaluating the same cost elements were used, and country differences can also provide reasoning to some extent. There were only a few studies that estimated the total health care costs associated with schizophrenia in a country, considering the population and prevalence of the disease in the country, and even less evaluating the indirect costs.

We found three systematic reviews with broadly similar scope to our work. All the three systematic literature reviews identified to be similar in context to our study used EMBASE and Medline as a primary database,^32–34^ while two also included PsycINFO. Zhao et al.^34^ focused on indirect costs but was concerned with comparing indirect costs between four selected chronic diseases, namely, asthma, diabetes, rheumatoid arthritis, and schizophrenia. The study conducted by Chong et al.^32^ gives detailed information on the methodological issues of COI studies and presents only aggregated data that does not include cost per patient. Our study is more focused towards European data, and we were concerned with the indirect costs only.

The most recently published systematic literature review was conducted by Jin et al.^33^ It was concerned by giving a general overview of COI studies for schizophrenia, and by comparing the societal cost of schizophrenia across countries. It also aimed to identify the main cost components of schizophrenia and factors associated with the higher societal cost to improve the quality and reporting of COI studies for schizophrenia.

When assessing the productivity loss calculation methods, the willingness-to-pay (WTP) methodology did not appear in the studies included in this review. Three studies applied the human capital approach (HCA), three studies applied friction cost approach (FCA) methodologies for the cost calculations, and two used other methodologies.

Cost of informal care (i.e. cost borne by caregivers) has been shown to be higher than the cost of productivity loss borne by schizophrenic patients themselves. In the study of Barnes et al.,^22^ cost of informal care was much higher than the cost of absenteeism, even though study results were not statistically significant. Frey validated the direction of the findings with high statistical significance, however, the magnitude of the difference was not as high.^28^ Informal care costs were more than two times the productivity loss borne by schizophrenic patients, according to Frey. Pletscher et al.^30^ reported the caregiver cost to be significantly lower. Again, the difference can be attributed to methodological differences, as Pletscher et al. used the average per capita production in Switzerland when unemployment, retirement, children, and part-time work are considered to value informal care and at the same time used the average monthly full-time gross wage plus 10% social security contributions by employers to account for productivity loss by patients themselves. Articles considering one indirect cost domain tend to report lower total indirect costs. According to Tajima-Pozo et al.,^19^, the real number of people affected by schizophrenia is much bigger than just the number of the patients.

The ratio of indirect costs to total cost varied significantly in the assessed papers which can be attributed to the aforementioned reasons of differences in indirect costs. The estimated average share of indirect costs (calculated to be 44%) most likely underestimates the real value of indirect cost percentage since many studies consider only productivity loss by patients themselves or by caregivers only. As stated by Rupp et al.,^35^ the direct and indirect costs are roughly equal, while other authors suggest that the indirect costs can be three to four times higher than the direct costs.^36^^,^^37^ Direct costs are usually well documented, and more details are considered during their calculations. For example, Sicras-Mainar et al.^31^ broke down direct costs into seven categories while considering only a single type of indirect costs.

Several studies indicated that different treatment regimens affect the total cost of schizophrenia as well as indirect costs. According to Einarson et al.,^25^ different treatment sequences using haloperidol, olanzapine, paliperidone, and risperidone in different dosage forms varied the total costs from 189 696–249 422 Swedish Krona, equivalent to 20 365–26 777 Euros, while the proportion of indirect costs varied from 2.4–3.8% of the total costs.

In Frey (2014),^28^ there was a significant variation in the productivity loss by patients themselves attributable to schizophrenia by age. Although the number of sick-leave days peaked among the 26–45 year-old patients (24.7 days) and sharply decreased with age (9.7 and 0.3 days for patients aged 46–65 years and patients above 65 years, respectively), indirect cost tends to increase with age until 65 years, followed by an instant decline. This could associate with the method (friction cost approach) used for estimating productivity loss that resulted in higher wages for elder population approaching retirement, and the decline beyond retirement. On the other hand, informal care costs followed an opposite trend, as patients not older than 25 years bared higher informal care costs (11% higher than average). Based on the reviewed studies both indirect cost elements (productivity loss by the patients and informal care) showed an overall minor increase of total indirect cost by aging.

According to Ekman et al.,^26^ male patients with schizophrenia face about 22% higher indirect costs than female patients, with a statistically significant difference. It can be attributed to the difference in employment rates and wages between genders.

Ekman et al. also showed that indirect costs are strongly related to the global assessment function (GAF) score of the patient, suggesting that attempts to improve global functioning by means of effective treatment might reduce the cost of schizophrenia. Patients with mild to serious symptoms (GAF score between 50–69) have 103% (15 207 Euros annually) higher indirect costs compared to patients with no or slight symptoms (GAF score is higher or equal to 70). This difference is even more significant comparing patients with serious symptoms to severe impairment (GAF score below 50) to patients with no or slight symptoms.

Results showed that negative symptoms are still considered controversial due to difficulty in defining and measuring them, as well as designing specific clinical trials for negative symptoms of schizophrenia.^38^^,^^39^ In most of the European countries, there are currently no pharmacological agents approved specifically for the treatment of negative symptoms,^40^ though the evaluation of two separable components of the negative symptom construct allows for the potential to evaluate differential efficacy of new therapeutic approaches.^41^ In a Spanish study, Sicras-Mainar et al.^31^ found one or more negative symptoms in 52.5% of patients with schizophrenia. Negative symptoms are reported to be better predictors of functioning than positive symptoms; however, the evidence on the impact of negative symptoms on healthcare costs related to schizophrenia is still scarce. According to the aforementioned study, patients with negative symptoms of schizophrenia had about 21% higher non-healthcare costs (productivity loss) over a period of 12 months, compared to patients without negative symptoms but the difference was not statistically significant. On the other hand, the difference in total cost (23%) between the negative and positive sub-groups was statistically significant.

## Limitations

The review was limited to articles written in English, and we excluded non-English articles, published since 2011 as our intention was to focus on the most recent evidence available. Different studies accounted for different indirect cost elements and even used different methodologies for quantification. Due to methodological heterogeneity of studies included in the review, focus was given to the qualitative analysis. Moreover, we referred to the data presented in the studies and did not perform a database search to specify more accurate prevalence data.

As Pletscher *et al.*^30^ evaluated presenteeism which was not a part of the search term, searching presenteeism systematically might have resulted in including more relevant papers, still we do not expect that the effect on the study findings would be substantial as those papers evaluating the cost of presenteeism are expected to evaluate other indirect cost items which were already included in the search terms. On this way, we believe that this limitation does not cause significant bias in the study conclusions.

Due to differences in methodology, cost values from two studies were not presented in the result section (i.e. table 2). Cortesi et al.^23^ reported only the number of days per patient-month of total productivity loss for both patients and caregivers, without quantifying the monetary value of the days lost. The study published by Crawford et al.^24^ solely considered the costs of criminal justice services related to crimes committed by patients with schizophrenia (i.e. prison, police custody, and probation officer services).

Two papers listed informal care (e.g. productivity loss tied to care by relatives) among direct non-medical costs.^28^^,^^30^ Based on the IQWiG description (see Method section) we re-categorized these cost elements to be included as indirect costs.

For four papers, calculating the share of indirect cost was not possible either because total cost was not reported,^21^^,^^29^ or the paper did not report indirect costs in monetary terms at all,^23^ or indirect cost components were too specific.^24^

There were several reasons to exclude potentially good candidates from the final analysis. Barnes et al. (2016) reported several barriers that led to the final sample size falling well short of the target recruitment of 358 participants. The authors, therefore, acknowledged that the power of any statistical analysis to detect clinically or statistically meaningful significant differences between the treatment arms in the study was limited. Einarson et al. (2014) reported indirect costs related to different treatment strategies, without presenting an average, so the average of the costs among different treatment scenarios was used for the calculations, assuming equal market share.

## Funding

We gratefully acknowledge the financial support of Gedeon Richter plc to conduct the systematic literature review.

*Conflicts of interest*: The work has been done with the help of the authors, Frank-Ulrich Fricke, PhD, MBA, Professor at Technische Hochschule Nürnberg, Germany who provided writing assistance for this article. Editorial assistance in formatting, proof reading, and copy editing were provided by Syreon Research Institute. Gedeon Richter provided funding to Syreon Research Institute for support in writing, and editing this article. The interpretation of the data was made by the authors independently. Ahmad Fasseeh is a part-time employee of Syreon Research Institute; Bertalan Németh is a full-time employee of Syreon Research Institute; Anett Molnár is a full-time employee of Syreon Research Institute; Margit Horváth is a full-time employee of Gedeon Richter; Kristóf Kóczián is a full-time employee of Gedeon Richter; Árpád Götze is a full-time employee of Gedeon Richter; Zoltan Kalo is a shareholder and the managing director of Syreon Research Institute.

**Key points**

- Societal perspective should be applied in the value judgment of new technologies in schizophrenia.
- Future cost of illness studies focusing on schizophrenia should aim at harmonizing their approaches.
- The average proportion of indirect cost was 44%, based on our review.
- Several factors influence the value of indirect costs of schizophrenia, including gender, age, the severity of the disease, the presence of negative symptoms, as well as the treatment regimen.
- More studies are needed to evaluate the indirect cost of schizophrenia in the Central, Eastern, and Southern regions of Europe. Reference 41 has been provided in Supplementary data.

## Supplementary Material

Supplementary Data

## References

[1] Frankenburg FR, Medscape, drugs & diseases, schizophrenia. Available at: https://emedicine.medscape.com/article/288259-overview (10 July 2017, date last accessed).

[2] Saarni SI, Viertiö S, Perälä J, et al Quality of life of people with schizophrenia, bipolar disorder and other psychotic disorders. Br J Psychiatry 2010;197:386–94. 21037216 10.1192/bjp.bp.109.076489

[3] McGrath J, Saha S, Chant D, Welham J Schizophrenia: a concise overview of incidence, prevalence, and mortality. Epidemiol Rev 2008;30:67–76. 18480098 10.1093/epirev/mxn001

[4] Wittchen H-U, Jacobi F, Rehm J, et al The size and burden of mental disorders and other disorders of the brain in Europe 2010. Eur Neuropsychopharmacol 2011;21:655–79. 21896369 10.1016/j.euroneuro.2011.07.018

[5] Penn DL, Kohlmaier JR, Corrigan PW Interpersonal factors contributing to the stigma of schizophrenia: social skills, perceived attractiveness, and symptoms. Schizophr Res 2000;45:37–45. 10978871 10.1016/s0920-9964(99)00213-3

[6] Mancuso A, Specchia ML, Lovato E, et al Economic burden of schizophrenia: the european situation. A scientific literature reviewAgostino Mancuso. Eur J Public Health 2014;24(suppl_2):352.24642604

[7] Koopmanschap MA, Rutten FF The impact of indirect costs on outcomes of health care programs. J Health Econ 1994;3:385–93. 10.1002/hec.4730030606 9435921

[8] Neumann PJ Costing and perspective in published cost-effectiveness analysis. Med Care 2009;47:S28–32.:19536023 10.1097/MLR.0b013e31819bc09d

[9] Schulz R, Beach SR Caregiving as a risk factor for mortality: the caregiver health effects study. JAMA 1999;282:2215–9. 10605972 10.1001/jama.282.23.2215

[10] Family caregiver alliance, caregiver statistics: work and caregiving-definitions. Available at: https://www.caregiver.org/caregiver-statistics-work-and-caregiving (4 October 2017, date last accessed).

[11] Van den Berg B, Brouwer WB, Koopmanschap MA Economic valuation of informal care. Eur J Health Econ, formerly: HEPAC 2004;5:36–45. 10.1007/s10198-003-0189-y 15452763

[12] Husereau D, Drummond M, Petrou S, et al Consolidated health economic evaluation reporting standards (CHEERS)—explanation and elaboration: a report of the ISPOR health economic evaluation publication guidelines good reporting practices task force. Value Health 2013;16:231–50. 23538175 10.1016/j.jval.2013.02.002

[13] Moher D, Shamseer L, Clarke M, et al Preferred reporting items for systematic review and meta-analysis protocols (PRISMA-P) 2015 statement. Syst Rev 2015;4:1. 25554246 10.1186/2046-4053-4-1 PMC4320440

[14] Larg A, Moss JR Cost-of-illness studies. Pharmacoeconomics 2011;29:653–71. 21604822 10.2165/11588380-000000000-00000

[15] World Bank, Consumer price index. Available at: https://data.worldbank.org/indicator/FP.CPI.TOTL (10 July 2017, date last accessed).

[16] OFX, Yearly Average Rates. Available at: https://www.ofx.com/en-gb/forex-news/historical-exchange-rates/yearly-average-rates/ (10 July 2017, date last accessed)

[17] Cost Estimation: Institut für Qualität und Wirtschaftlichkeit im Gesundheitswesen (IQWiG); 2009 Nov. Working Paper: Version 1.0–19/11/2009.

[18] Koopmanschap MA, Rutten FF, van Ineveld BM, Van Roijen L The friction cost method for measuring indirect costs of disease. J Health Econ 1995;14:171–89. 10154656 10.1016/0167-6296(94)00044-5

[19] Tajima-Pozo K, de Castro Oller MJ, Lewczuk A, Montañes-Rada F Understanding the direct and indirect costs of patients with schizophrenia. F1000Res 2015;4:182. Doi: 10.12688/f1000research.6699.2. 26339474 10.12688/f1000research.6699.1 PMC4544407

[20] McGuffin P, Farmer A, Harvey I A polydiagnostic application of operational criteria in studies of psychotic illness: development and reliability of the OPCRIT system. Arch Gen Psychiatry 1991;48:764–70. 1883262 10.1001/archpsyc.1991.01810320088015

[21] Aranda-Reneo I, Oliva-Moreno J, Vilaplana-Prieto C, et al Informal care of patients with schizophrenia. J Ment Health Policy Econ 2013;16:99–108. 24327480

[22] Barnes TR, Leeson VC, Paton C, et al Antidepressant controlled trial for negative symptoms in Schizophrenia (ACTIONS): a double-blind, placebo-controlled, randomised clinical trial. Health Technol Assess 2016;20:1–46. 10.3310/hta20290 PMC4860560 27094189

[23] Cortesi PA, Mencacci C, Luigi F, et al Compliance, persistence, costs and quality of life in young patients treated with antipsychotic drugs: results from the COMETA study. BMC Psychiatry 2013;13:98. 23522406 10.1186/1471-244X-13-98 PMC3621844

[24] Crawford MJ, Helen K, Barnes Thomas RE, Barrett B, et al Group art therapy as an adjunctive treatment for people with schizophrenia: a randomised controlled trial (MATISSE. ). Health Technol Assess 2012;16:1–76. 10.3310/hta16080 22364962

[25] Einarson TR, Vicente C, Zilbershtein R, et al Pharmacoeconomics of depot antipsychotics for treating chronic schizophrenia in Sweden. Nord J Psychiatry 2014;68:416–27. 24274837 10.3109/08039488.2013.852243

[26] Ekman M, Granström O, Omérov S, et al The societal cost of schizophrenia in Sweden. J Ment Health Policy Econ 2013;16:13–25. 23676412

[27] Evensen S, Wisløff T, Lystad JU, et al Prevalence, employment rate, and cost of schizophrenia in a high-income welfare society: a population-based study using comprehensive health and welfare registers. Schizophr. Bull 2016;42:476–83. 10.1093/schbul/sbv141 PMC4753607 26433216

[28] Frey S The economic burden of schizophrenia in Germany: a population-based retrospective cohort study using genetic matching. Eur Psychiatry 2014;29:479–89. 24853296 10.1016/j.eurpsy.2014.04.003

[29] Gupta S, Isherwood G, Jones K, Van Impe K Productivity loss and resource utilization, and associated indirect and direct costs in individuals providing care for adults with schizophrenia in the EU5. Clinicoecon Outcomes Res 2015;7:593–602. 26648745 10.2147/CEOR.S94334 PMC4664428

[30] Pletscher M, Mattli R, von Wyl A, et al The societal costs of schizophrenia in Switzerland. J Ment Health Policy Econ 2015;18:93–103. 26231000

[31] Sicras-Mainar A, Maurino J, Ruiz-Beato E, Navarro-Artieda R Impact of negative symptoms on healthcare resource utilization and associated costs in adult outpatients with schizophrenia: a population-based study. BMC Psychiatry 2014;14:225. 25096022 10.1186/s12888-014-0225-8 PMC4149268

[32] Chong HY, Teoh SL, Wu DBC, et al Global economic burden of schizophrenia: a systematic review. Neuropsychiatr Dis Treat 2016;12:357–73. 26937191 10.2147/NDT.S96649 PMC4762470

[33] Jin H, Mosweu I The societal cost of schizophrenia: a systematic review. Pharmacoeconomics 2017;35:25–42. 27557994 10.1007/s40273-016-0444-6

[34] Zhao FL, Xie F, Hu H, Li SC Transferability of indirect cost of chronic disease: a systematic review and meta-analysis. Pharmacoeconomics 2013;31:501–8. 23620212 10.1007/s40273-013-0053-6

[35] Rupp A, Keith SJ The costs of schizophrenia: assessing the burden. Psychiatr Clin North Am 1993;16:413–23. 8332569

[36] Andrews G, Sanderson K, Corry J, et al Cost-effectiveness of current and optimal treatment for schizophrenia. Br J Psychiatry 2003;183:427–35. 14594918 10.1192/bjp.183.5.427

[37] Davies LM, Drummond MF Economics and schizophrenia: the real cost. Br J Psychiatry Suppl 1994;165(S25);18–21. 7865193

[38] Daniel DG Issues in selection of instruments to measure negative symptoms. Schizophr Res 2013;150:343–5. 23899996 10.1016/j.schres.2013.07.005

[39] Marder SR, Alphs L, Anghelescu I-G, et al Issues and perspectives in designing clinical trials for negative symptoms in schizophrenia. Schizophr Res 2013;150:328–33. 24028744 10.1016/j.schres.2013.07.058

[40] Arango C, Garibaldi G, Marder SR Pharmacological approaches to treating negative symptoms: a review of clinical trials. Schizophr Res 2013;150:346–52. 23938176 10.1016/j.schres.2013.07.026

