# Supplementary material for: A systematic review of the indirect costs of schizophrenia in Europe
Source: Eur J Public Health. 2018 Nov 5;28(6):1043–9. doi: 10.1093/eurpub/cky231 (PMC6241204; doi:10.1093/eurpub/cky231)
Supplement: Supplementary Data [file cky231_supplementary_appendix.doc]

# Appendix

| **Database** | **Search strategy** | **Date of search** | **Hit Number** |
| --- | --- | --- | --- |
| **Scopus** | TITLE-ABS-KEY ( ( "Schizophrenia"  OR  "Schizophrenic"  OR  "Bleuler's disease"  OR  "hebephrenia"  OR  "paranoia"  OR  "shared paranoid disorder"  OR  "psychotic affective disorder"  OR  "hebephrenic disorder"  OR  "psychoaffective disorder" )  AND  ( "Productivity"  OR  "Societal cost"  OR  "Time off"  OR  "Caregiver"  OR  "Absenteeism"  OR  "Days off"  OR  "Social cost"  OR  "Sick leave"  OR  "Absence"  OR  "indirect costs"  OR  "indirect cost" ) )  AND  ( LIMIT-TO ( PUBYEAR ,  2017 )  OR  LIMIT-TO ( PUBYEAR ,  2016 )  OR  LIMIT-TO ( PUBYEAR ,  2015 )  OR  LIMIT-TO ( PUBYEAR ,  2014 )  OR  LIMIT-TO ( PUBYEAR ,  2013 )  OR  LIMIT-TO ( PUBYEAR ,  2012 )  OR  LIMIT-TO ( PUBYEAR ,  2011 ) )  AND  ( LIMIT-TO ( LANGUAGE ,  "English " ) ) | 30.03.2017 | 1,613 |
| **Ovid** | 1-(("Schizophrenia" or "Schizophrenic" or "Bleuler's disease" or "hebephrenia" or "paranoia" or "shared paranoid disorder" or "psychotic affective disorder" or "hebephrenic disorder" or "psychoaffective disorder") AND ("Productivity" or "Societal cost" or "Time off" or "Caregiver" or "Absenteeism" or "Days off" or "Social cost" or "Sick leave" or "Absence" or "indirect cost" or "indirect costs")).mp. [mp=title, abstract, heading word, table of contents, key concepts, original title, tests & measures]  Limit 1 to (English language and yr=”2011”) | 30.03.2017 | 126 |
